# Trigger Point Dry Needling and Proprioceptive Exercises for the Management of Chronic Ankle Instability: A Randomized Clinical Trial

**DOI:** 10.1155/2015/790209

**Published:** 2015-04-30

**Authors:** Jaime Salom-Moreno, Blanca Ayuso-Casado, Beatriz Tamaral-Costa, Zacarías Sánchez-Milá, César Fernández-de-las-Peñas, Francisco Alburquerque-Sendín

**Affiliations:** ^1^Department of Physical Therapy, Occupational Therapy, Rehabilitation and Physical Medicine, Universidad Rey Juan Carlos, Avenida de Atenas s/n, Alcorcón, 28922 Madrid, Spain; ^2^Cátedra de Investigación y Docencia en Fisioterapia: Terapia Manual y Punción Seca, Universidad Rey Juan Carlos, Avenida de Atenas s/n, Alcorcón, 28922 Madrid, Spain; ^3^Grupo de Excelencia Investigadora URJC-Banco de Santander: Investigación Traslacional en el Proceso de Salud-Enfermedad (ITPSE), Avenida de Atenas s/n, Alcorcón, 28922 Madrid, Spain; ^4^Centro de Fisioterapia CRENE, Caunedo 24, 28037 Madrid, Spain; ^5^Department of Physical Therapy, Universidad de Salamanca, Avenida Donante de Sangre s/n, 37007 Salamanca, Spain

## Abstract

*Objective.* To compare the effects of combined trigger point dry needling (TrP-DN) and proprioceptive/strengthening exercises to proprioceptive/strengthening exercises on pain and function in ankle instability.* Methods.* Twenty-seven (44% female, mean age: 33 ± 3 years) individuals with unilateral ankle instability were randomly assigned to an experimental group who received proprioceptive/strengthening exercises combined with TrP-DN into the lateral peroneus muscle and a comparison group receiving the same proprioceptive/strengthening exercise program alone. Outcome included function assessed with the Foot and Ankle Ability Measure (FAAM) and ankle pain intensity assessed with a numerical pain rate scale (NPRS). They were captured at baseline and 1-month follow-up after the intervention.* Results.* The ANOVAs found significant Group ∗ Time Interactions for both subscales of the FAAM (ADL: *F* = 8.211; *P* = 0.008; SPORTS: *F* = 13.943; *P* < 0.001) and for pain (*F* = 44.420; *P* < 0.001): patients receiving TrP-DN plus proprioceptive/strengthening exercises experienced greater improvements in function and pain than those receiving the exercise program alone. Between-groups effect sizes were large in all outcomes (SMD > 2.1) in favor of the TrP-DN group.* Conclusions.* This study provides evidence that the inclusion of TrP-DN within the lateral peroneus muscle into a proprioceptive/strengthening exercise program resulted in better outcomes in pain and function 1 month after the therapy in ankle instability.

## 1. Introduction

Lateral ankle sprain is the most common form of ankle sprain experienced by subjects participating in athletic activities and results in substantial societal burden [1]. Lateral ankle sprain can be a single injury event or part of an ongoing process that leads to functional ankle instability. In fact, up to 40% of ankle sprains can result in chronic ankle instability [2, 3]. An overall prevalence of chronic ankle instability of 1.1% has been recently reported in males and 0.7% in females in a large general population study [4]. Tanen et al. found a prevalence rate of 23% of ankle instability in high school athletes [5].

Ankle instability symptoms include subjective feeling of ankle giving way, pain, swelling, resprain, and/or limitations in daily life activities and sports participation [6]. Chronic ankle instability can be developed by several and different contributing factors including mechanical (joint laxity, altered kinematics, and degenerative or synovial changes to the cartilage of the talocrural joint) or functional (deficits in proprioception, strength, or muscular control) impairments [7].

Conservative management is the initial therapeutic option for these patients; however, the most appropriate strategies are still unclear. Recent studies support the use of joint mobilization techniques for the management of ankle instability symptoms [8–10]. Nevertheless, it seems that neuromuscular/proprioceptive interventions are the most appropriate therapeutic tools for the treatment of this condition. The Cochrane review concluded that neuromuscular training was effective at a short-term for chronic ankle instability compared to no training [11]. A recent systematic review concluded that a training program gives better results for pain and function, and a decrease of recurrent ankle sprains, than a wait-and-see policy in individuals with chronic ankle instability; however, the clinical evidence for this effect exhibited limited to moderate level [12].

The role of surrounding soft tissues in the aetiology of chronic ankle instability is based on alterations in soft tissue function that may occur in the surrounding muscles. A recent meta-analysis determined that subjects presenting with ankle instability exhibit delayed peroneal reaction time when compared with the contralateral uninvolved limb or with healthy controls [13]. This evidence supports the presence of muscle control disturbances in the surrounding musculature in patients with ankle instability. Repetitive ankle injuries are proposed as one potential mechanism for activation of trigger points (TrPs) [14]. TrPs have been related to the presence of altered motor control patterns [15] and accelerated muscle fatigability [16] in the affected and related musculature. Therefore, proper treatment of TrPs may effectively reduce these motor disturbances in the affected musculature by preventing overload spreading on the surrounding structures [15, 16]. Some authors have claimed that trigger point dry needling (TrP-DN) is an effective therapeutic approach for the management of sensory and motor components of TrPs [17].

No studies to date have examined the efficacy of including TrP-DN combined with a proprioceptive/strengthening program in subjects with chronic ankle instability. Therefore, the purpose of this randomized clinical trial was to compare the effects of combined TrP-DN and proprioceptive/strengthening exercise program to proprioceptive/strengthening program alone on pain and function in individuals with chronic ankle instability. We hypothesized that those individuals receiving TrP-DN combined with proprioceptive/strengthening exercise program will exhibit higher improvements in pain and function than individuals receiving the proprioceptive and strengthening program alone.

## 2. Methods

### 2.1. Participants

Consecutive individuals with unilateral ankle instability presenting to a physical therapy clinic in Madrid (Spain) from January 2013 to June 2014 were screened for inclusion in this study. To be included in the study patients had to fulfil the following criteria: (1) age between 18 and 50 years, (2) history of at least one ankle sprain, (3) at least one episode of giving away in the previous 6 months, (4) ankle pain of intensity > 3 points on an 11-point numerical pain rate scale (NPRS), and (5) score of 25 or less on the Cumberland Ankle Instability Tool [18, 19]. In addition, participants also had to be physically active, defined as participating in vigorous physical activity at least 20 min a day, 3 times a week [20]. Participants were allowed to continue their regular physical activities during the study period.

Participants were excluded if exhibited any of the following criteria: (1) fracture in the lower extremity, (2) history of surgery in the lower extremity, (3) any concomitant lower extremity pathology, for example, vascular disease and osteoarthritis, (4) pregnancy, (5) regular use of medication, or (6) previous physical therapy interventions received on the lower extremity within the previous 6 months. The study protocol was approved by the Ethical Committee of the Universidad Rey Juan Carlos and it was conducted according to the Helsinki Declaration. All participants read and signed an informed consent prior to their inclusion in the study.

### 2.2. Outcome Measures

The primary outcome measure of this study was the Foot and Ankle Ability Measure (FAAM). A recent systematic literature has found that the FAAM is the most commonly used outcome measure for assessing function in the lower extremity [21]. The FAAM is a 29-item questionnaire divided into 2 scales: (1) activities of daily living (ADL) subscale including 21 items and (2) sports (SPORTS) subscale including 8 items. Each item is scored on a 5-point Likert (0–4) scale representing different levels of difficulty. Values are summed for calculating the score of each scale, 84 points for the ADL and 32 points for SPORTS scale [22]. Either score is transformed to percentage (0–100%) to get the final score of each subscale with higher scores indicating a higher functional status. Test-retest reliability is high, 0.89 for ADL and 0.87 for SPORTS subscale. In fact, the FAAM has shown to be valid for its use in patients with ankle instability [23].  It has been suggested that the minimal clinically important difference (MCID) was 8 and 9 points for the ADL and SPORTS subscales, respectively [22].

In the secondary outcome, the intensity of ankle pain during sport practicing was assessed with an 11-point numerical pain rating scale (NPRS), where 0 is the absence of pain and 10 represents maximum pain [24]. There is no available data for the MCID for patients with ankle instability; nevertheless, it seems that changes ranging between 1.5 and 2.1 points can be considered as the MCID score for patients with musculoskeletal pain conditions [25, 26].

Outcomes were captured at baseline and at 1 month after the last treatment session.

### 2.3. Randomization

Following the baseline examination, patients were randomly assigned to receive proprioceptive/strengthening exercise program alone (control group) or proprioceptive/strengthening exercise program combined with TrP-DN into the lateral peroneus muscle (experimental group). Concealed allocation was performed using a computer-generated randomized table of numbers created prior to data collection by an external researcher. Individual and sequentially numbered index cards with the random assignment were prepared. The index cards were folded and placed in sealed opaque envelopes. A second external researcher opened the envelope and proceeded with treatment according to the group assignment.

Each group was treated by a clinician with more than 10 years of experience in the management of lower extremity injuries. A systematic review has concluded that proprioceptive exercise programs are commonly delivered over a 6-week period, with a frequency varying between 1 and 7 times weekly and with a duration ranging from 10 min to 1 h [27]. Nevertheless, some authors suggest that proprioceptive programs may need to be completed over a longer time period, that is, eight weeks, for getting proper results. Therefore, in the current study both groups exercised twice a week for 8 weeks [28]. All subjects trained using their affected ankle only.

The experimental group also received TrP-DN in the lateral peroneus muscle of the affected extremity. TrP-DN was applied once per week for the first 4 weeks before starting any exercise on that session. Individuals were unaware of the real objective of the study in that they were aware of the clinical implications without revealing the real intervention that was being evaluated. All subjects were informed of the true nature of the study at the end of the trial.

### 2.4. Proprioceptive and Strengthening Exercise Program

Kim et al. have recently demonstrated that combination of muscle strengthening and proprioceptive exercises is more effective than only muscle strengthening exercises for the management of ankle instability [29]. The strengthening/proprioceptive training program applied in our trial was based on best-available evidence and common clinical practice. All exercises were performed under the supervision of the respective clinician.

The strengthening program included a protocol consisting of the use of Thera-Bands according to the protocol described by Kaminski et al. [30]. Participants sat on the floor with one end of the tubing tied around a treatment table and the other end around the metatarsal heads of the affected foot. Knees were fully extended, and the Thera-Band was stretched to 170% of its resting length, regardless of band color (resistance). Strengthening exercises included all movements of the ankle. The exercise progression involved an increased number of sets (1–3 sets of 8–10 repetitions each) or increased resistance each week depending on the symptomatology of the subject [31].

**Figure 1:**
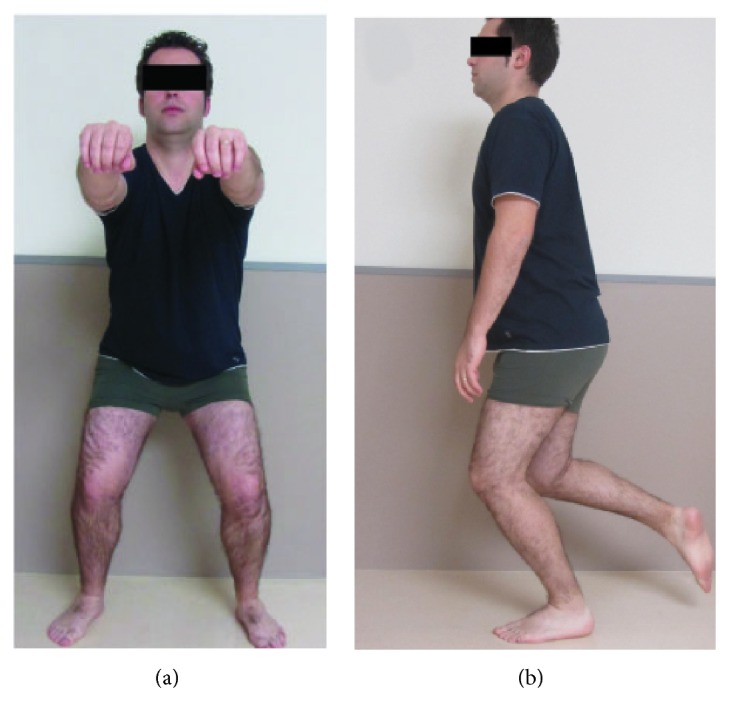
Closed kinetic chain exercises on stable surface: (a) bilateral semisquats; (b) one leg standing exercise.

**Figure 2:**
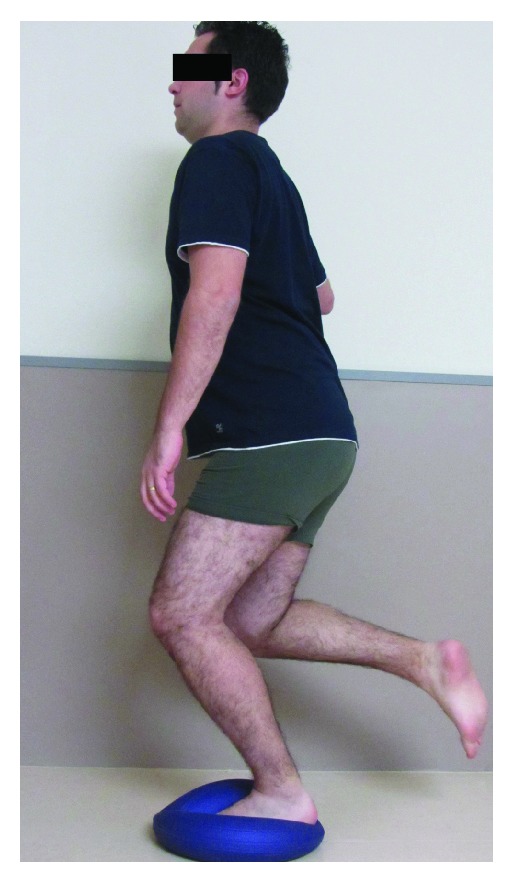
One leg standing exercise on unstable surface.

**Figure 3:**
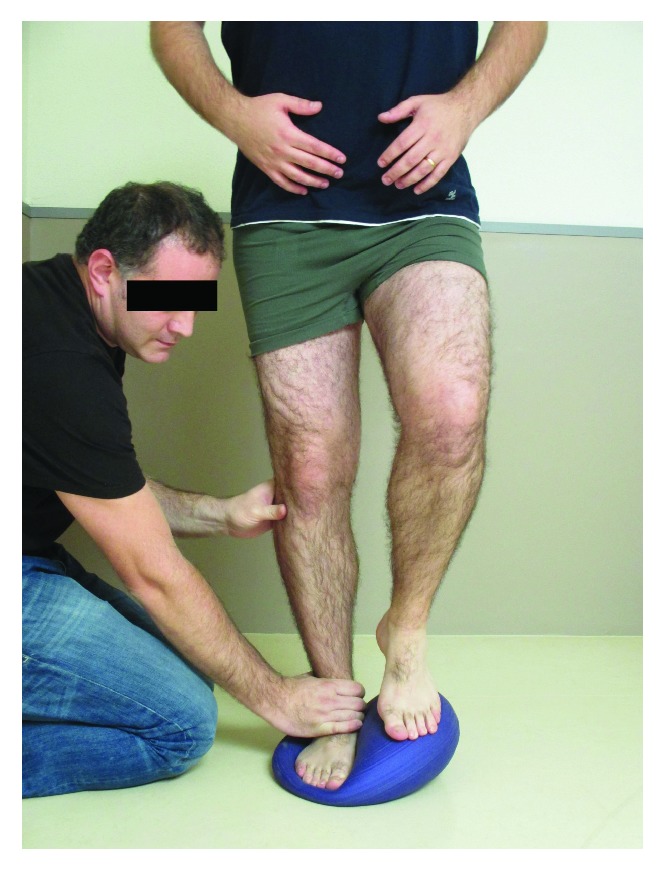
One leg standing exercise on unstable surface including perturbation training by the therapist.

The proprioceptive exercise program consisted of a number of closed kinetic chain exercises in weight bearing positions. The clinician reinforced the patient through progressive lower extremity loading from bilateral to unilateral load acceptance. The exercises applied in the current study consisted of semisquats (Figure 1(a)) and one leg standing exercise with eyes opened or closed (Figure 1(b)) on stable surface. Patients performed 3 sets of 10 repetitions of each exercise for the first 2 weeks. In the following weeks (weeks 3-4), the same exercises were progressed to unstable surfaces (Figure 2). Within the last 4 weeks (weeks 5–8) some perturbation training was included (Figure 3). All these exercises focus on motor control of eccentric contractions of the ankle muscles to boost this musculature for proper contribution to ankle stabilization [32].

### 2.5. Trigger Point Dry Needling (TrP-DN)

TrP-DN was applied to the lateral peroneus muscle by a clinician with more than 6 years of experience in the management of TrPs with this technique. Patients received TrP-DN with disposable stainless steel needles (0.3 mm × 30 mm, Novasan) that were inserted into the skin over the TrP area. TrP diagnosis was determined when all the following criteria were present [33]: (1) hypersensitive spot in a palpable taut band of the lateral peroneus muscle, (2) palpable or visible local twitch on pincer palpation, and (3) reproduction of referred pain elicited by palpation of the sensitive spot. The referred pain from the lateral peroneus muscle spreads to the lateral aspect of the ankle mimicking ankle sprain/instability pain [33]. These criteria have shown to exhibit good interexaminer reliability (kappa: 0.84–0.88) when are applied by an experienced clinician [34].

**Figure 4:**
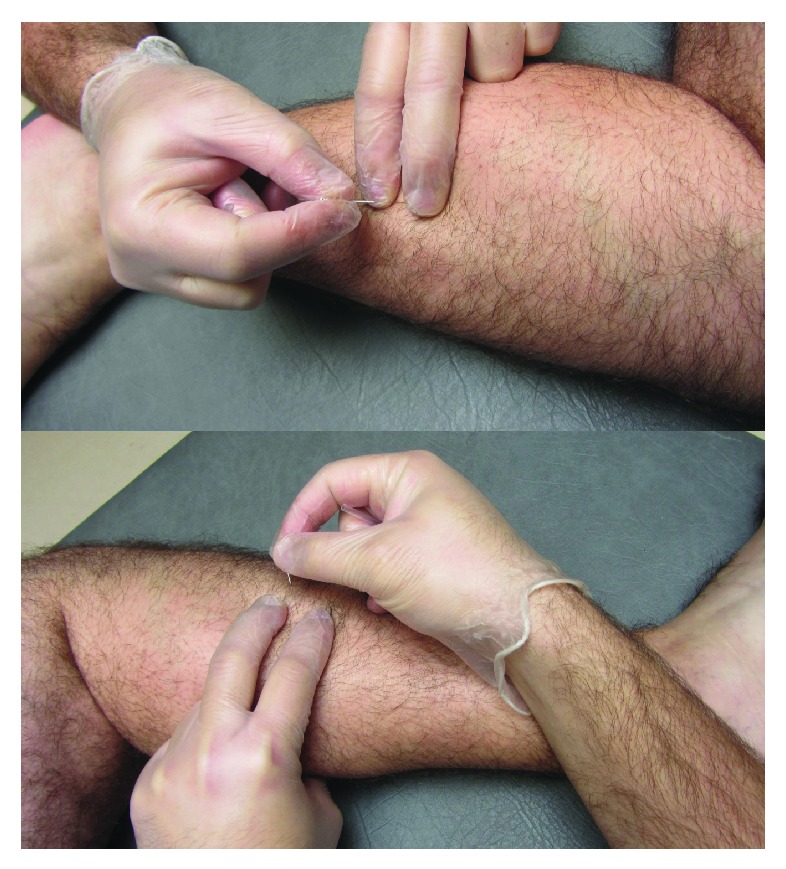
Trigger point dry needling (TrP-DN) applied over the lateral peroneus muscle. With the patient in side-lying position, the needle was inserted into the skin over the TrP until the first local twitch response was obtained and moved up and down (2 to 3 mm vertical motions with no rotations) at approximately 1 Hz for 25–30 seconds.

In this study, the fast-in and fast-out technique described by Hong was applied [35]. Once the TrP was located with flat palpation in the lateral peroneus muscle, the overlying skin was cleaned with alcohol. The needle was inserted, penetrating the skin 10–15 mm into the TrP until the first local twitch response was obtained (Figure 4). It is suggested that local twitch responses should be elicited during TrP-DN for a proper and successful technique [35]. Once the first local twitch response was obtained, the needling was moved up and down (2 to 3 mm vertical motions with no rotations) at approximately 1 Hz for 30–45 seconds.

### 2.6. Sample Size Calculation

Sample size and power calculations were performed with the ENE 3.0 software (GlaxoSmithKline, Universidad Autónoma, Barcelona, Spain). The calculations were based on detecting a mean difference of 8.0 points (MCID) on each subscale of the FAAM [22], assuming a standard deviation of 6.5, a 2-tailed test, an alpha level of 0.05, and a desired power of 90%. The estimated desired sample size was 12 participants per group.

### 2.7. Adverse Events

All participants were asked to report any adverse events that they experienced during all the study and the 1-month follow-up period. An adverse event was defined as sequelae of medium-term in duration with any symptom perceived as distressing and unacceptable to the individual and that required further treatment [36]. Since TrP-DN sometimes induces posttreatment soreness, subjects were advised to report any increase in their symptoms.

### 2.8. Statistical Analysis

Statistical analysis was performed using SPSS statistical software, version 18.0, and it was conducted according to the intention-to-treat analysis principle. Mean, standard deviation, and/or 95% confidence interval were calculated for each variable. The Kolmogorov-Smirnov test showed a normal distribution of all the data (*P* > 0.05). Baseline demographic and clinical variables between both groups were compared using independent Student's *t*-tests for continuous data and *χ* ^2^ tests of independence for categorical data. A 2 × 2 repeated measured ANOVA with time (baseline, 1 month after) as within-subjects factor and group (control or experimental) was used to calculate changes in the outcomes (pain and function). The main hypothesis of interest was the Group by Time Interaction at an a priori alpha-level equal to 0.05.

To enable comparison of effect sizes, standardized mean score differences (SMDs) were calculated by dividing the mean score differences between TrP-DN plus proprioceptive/strengthening exercise program and comparison group (proprioceptive/strengthening program alone) by the pooled standard deviation.

## 3. Results

**Figure 5:**
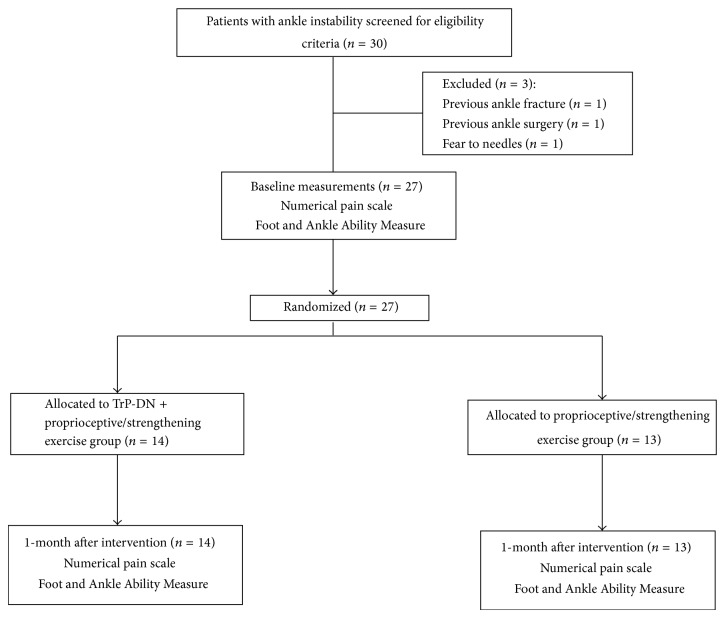
Flow diagram of patients throughout the course of the study.

Thirty consecutive individuals with ankle instability were screened for eligibility criteria. Twenty-seven patients (mean ± SD age: 33 ± 3 years; 44% female) satisfied the eligibility criteria, agreed to participate, and were randomized into TrP-DN experimental group (*n* = 14) or comparative group (*n* = 13). The reasons for ineligibility can be found in Figure 5, which provides a flow diagram of patient recruitment and retention. Baseline features between both groups were similar for all variables (Table 1).

The mixed model ANOVAs revealed a significant Group ∗ Time Interaction for both subscales of the FAAM (ADL: *F* = 8.211; *P* = 0.008; SPORTS: *F* = 13.943; *P* < 0.001): patients receiving TrP-DN plus proprioceptive/strengthening exercises experienced greater increase in function than those receiving proprioceptive/strengthening exercises program alone (Table 2). Between-groups effect sizes were large in both subscales (SMD > 2.11) in favour of the TrP-DN plus proprioceptive/strengthening exercise group. Table 2 provides baseline and 1-month data after intervention as well as within-group differences with their associated 95% CI for both FAAM subscales.

The 2 × 2 ANOVA also revealed a significant Group ∗ Time Interaction for ankle pain (*F* = 44.420; *P* < 0.001) with patients receiving the combination of TrP-DN and proprioceptive/strengthening exercises experiencing a greater reduction in pain intensity than those receiving proprioceptive/strengthening exercises program alone (Table 2). Between-groups effect size was large (*d* > 2.3) for the decrease in ankle pain in favour of the TrP-DN plus proprioceptive/strengthening exercises group.

In our study, 8 individuals (57%) assigned to the TrP-DN plus proprioceptive/strengthening exercises group experienced some muscle soreness at the lateral peroneus muscle after treatment but did not experience an increase of their ankle symptoms. TrP-DN posttreatment soreness resolved spontaneously within 24–36 hours without any intervention.

## 4. Discussion

The results of this randomized clinical trial suggest that the combination of TrP-DN plus proprioceptive/strengthening exercise program resulted in better outcomes 1 month after the end of therapy than when only proprioceptive/strengthening exercises were applied in individuals with chronic ankle instability. We could anticipate that the benefit of adding TrP-DN for the management of ankle instability could be clinically relevant as noted by the large between-group effect sizes in all the outcomes, although further studies are needed.

Different systematic reviews had concluded that manual therapies [37], exercises [38], or functional rehabilitation [39] result in improved outcomes in patients with ankle instability. On the contrary, ankle brace or ankle tape has no effect on proprioceptive acuity in subjects with ankle instability [40]. Those recommendations did not identify TrP-DN as a potential effective intervention for ankle instability not because there was evidence against this intervention but rather because there was a lack of quality studies on the topic. Our study is the first one investigating the effectiveness of TrP-DN on pain and function in individuals with chronic ankle instability. In fact, there is no available data on the prevalence of active TrPs in this pain population. It is interesting to note that all participants included within the experimental group exhibited active TrPs in the lateral peroneus muscle reproducing, at least, part of their symptoms.

We found that subjects with ankle instability who received TrP-DN in addition to a proprioceptive/strengthening exercise program resulted in higher improvements in function in both ADL and SPORTS subscales than those receiving only the exercise program. It should be noted that while between-groups change scores were statistically significant and surpassed the reported MCID for both subscales of the primary outcome (FAAM), the clinical significance of these differences is not as clear given the inclusion of the MCID within the 95% CIs for these comparisons (Table 2). This may be related to the fact that proprioceptive/strengthening exercise programs are clinically effective for management of ankle sprains [11, 38] and both groups received the same program. In fact, our results within the proprioceptive/strengthening exercise group were similar to previous studies where moderate effects on pain and function were also found [12]. Our results would further support the use of proprioceptive/strengthening exercises for improving function in patients with chronic ankle instability.

Our study also observed that individuals receiving TrP-DN exhibited a higher decrease in pain intensity than those who did not receive the intervention. In this case, between-group change scores surpassed the MCID for the outcome [25, 26] in favour of the TrP-DN group. Nevertheless, the data also indicate that individuals receiving the proprioceptive/strengthening exercise program alone also experienced statistically and clinically significant improvement in pain with the lower bound of the 95% confidence interval for within-group change score being equal than the MCID for pain intensity. It is possible that TrP-DN can help to decrease the pain in subjects with ankle instability.

The exact therapeutic mechanism by which TrP-DN exerts its effects remains to be elucidated [17]. Both mechanical [41] (e.g., disruption of the contraction knot or increase of sarcomere length) and neurophysiological [42] (i.e., decrease of peripheral nociception and activation of central pain pathways) mechanisms have been suggested. It is possible that the combination of several mechanisms resulted in the improved outcomes in pain and function [43]. For instance, restoration of the length of the muscle sarcomeres of the lateral peroneus may improve motor output of the muscle explaining the improvement in function, whereas the decrease in peripheral nociception could be related to the decrease in pain.

Because the addition of TrP-DN resulted in statistically and potentially clinically greater improvements in pain and function in individuals with ankle instability we may hypothesize that the ankle eversor muscles can play a relevant role and may perpetuate symptoms associated with ankle instability. In fact, a recent meta-analysis has supported the presence of delayed peroneal reaction time in subjects with ankle instability [13]. It is plausible that TrP-DN applied on the lateral peroneus muscles before the beginning of proprioceptive/strengthening exercises can improve the motor output of this muscle [17, 43]. Future studies are now needed to further determine the motor effects of TrP-DN.

Finally, we should recognize that a number of limitations existed in the current study. First, only 1 therapist provided the treatment to each group, respectively, which may limit the generalizability of the results. Second, we only assessed outcomes at 1-month follow-up and cannot be certain if these differences remained in the long term. Third, we did not also assess the perspective of the patients about the progress of their instability by using a self-reported method of evaluation like the Global Rating of Change (GROC). Finally, the influence of a placebo effect is unknown as we did not include a group receiving a sham intervention [44, 45] and we did not evaluate the real blinding of participants by a questionnaire at the end of the study. Future randomized clinical trials should include multiple therapists delivering the interventions, a sham-control group, and long-term follow-up.

## 5. Conclusion

This study provides evidence that the inclusion of TrP-DN within the lateral peroneus muscle into a proprioceptive/strengthening exercise program resulted in better outcomes in pain and function 1 month after the end of the therapy in individuals with ankle instability. Our results may anticipate that the benefits of adding TrP-DN in the lateral peroneus muscle for the management of ankle instability are clinically relevant as large between-groups effect sizes were observed in all the outcomes. Future studies should include a control group and examine the long-term effects of these interventions in this population.

## Figures and Tables

**Table 1:** Baseline demographics for both groups.

	Proprioceptive/strengthening (*n* = 13)	TrP-DN + proprioceptive/strengthening (*n* = 14)	*P* value
Gender (male/female)	7/6	8/6	*χ* ^2^ = 0.30; *P* = 0.863
Age (years)	33.4 ± 2.8	33.0 ± 2.4	*t* = 0.524; *P* = 0.605
Months with instability of the condition	8.9 ± 1.3	9.2 ± 1.8	*t* = −0.360; *P* = 0.722
Cumberland Ankle Instability Tool score	18.2 ± 2.4	17.9 ± 2.1	*t* = 0.353; *P* = 0.727
FAAM-ADL subscale (0–100)	87.3 ± 5.9	83.9 ± 8.2	*t* = 1.227; *P* = 0.231
FAAM-SPORTS subscale (0–100)	73.0 ± 13.9	71.6 ± 16.4	*t* = 0.242; *P* = 0.811
Ankle pain intensity (0–10)	5.5 ± 0.9	5.8 ± 1.1	*t* = −0.832; *P* = 0.413

Values are expressed as mean ± standard deviation; FAAM: Foot and Ankle Ability Measure.

**Table 2:** Baseline, final treatment session, and change scores for FAAM subscales and pain intensity.

Outcome group	Baseline	End of treatment	Within-group change scores	Between-group difference in change scores
FAAM-ADL subscale (0–100)				
Proprioceptive/strengthening	87.3 ± 5.9	90.6 ± 7.2	3.3 (−1.4, 7.8)	8.2 (2.3, 14.1)
TrP-DN + proprioceptive/strengthening	83.9 ± 8.2	95.4 ± 5.5	11.5 (7.3, 15.6)	

FAAM-SPORTS subscale (0–100)				
Proprioceptive/strengthening	73.0 ± 13.9	81.0 ± 10.4	8.0 (4.0, 12.1)	12.1 (5.5, 18.9)
TrP-DN + proprioceptive/strengthening	71.6 ± 16.4	91.7 ± 9.0	20.1 (14.5, 25.9)	

Ankle pain intensity (0–10)				
Proprioceptive/strengthening	5.5 ± 0.9	3.5 ± 0.8	−2.0 (−2.5, −1.5)	2.4 (1.8, 3.1)
TrP-DN + proprioceptive/strengthening	5.8 ± 1.1	1.4 ± 1.0	−4.4 (−4.9, −4.0)	

Values are expressed as mean ± standard deviation for baseline and final means and as mean (95% confidence interval) for within- and between-group change scores.

FAAM: Foot and Ankle Ability Measure.
